# Structural descriptor database: a new tool for sequence-based functional site prediction

**DOI:** 10.1186/1471-2105-9-492

**Published:** 2008-11-25

**Authors:** Juliana S Bernardes, Jorge H Fernandez, Ana Tereza R Vasconcelos

**Affiliations:** 1 Laboratório Nacional de Computação Científica LNCC/MTC, Rua Getúlio Vargas 333, Quitandinha, Petrópolis, RJ, 25651-070, Brazil

## Abstract

**Background:**

The Structural Descriptor Database (SDDB) is a web-based tool that predicts the function of proteins and functional site positions based on the structural properties of related protein families. Structural alignments and functional residues of a known protein set (defined as the training set) are used to build special Hidden Markov Models (HMM) called HMM descriptors. SDDB uses previously calculated and stored HMM descriptors for predicting active sites, binding residues, and protein function. The database integrates biologically relevant data filtered from several databases such as PDB, PDBSUM, CSA and SCOP. It accepts queries in fasta format and predicts functional residue positions, protein-ligand interactions, and protein function, based on the SCOP database.

**Results:**

To assess the SDDB performance, we used different data sets. The Trypsion-like Serine protease data set assessed how well SDDB predicts functional sites when curated data is available. The SCOP family data set was used to analyze SDDB performance by using training data extracted from PDBSUM (binding sites) and from CSA (active sites). The ATP-binding experiment was used to compare our approach with the most current method. For all evaluations, significant improvements were obtained with SDDB.

**Conclusion:**

SDDB performed better when trusty training data was available. SDDB worked better in predicting active sites rather than binding sites because the former are more conserved than the latter. Nevertheless, by using our prediction method we obtained results with precision above 70%.

## Background

In the post-genomic era, functional identification of proteins and their interactions became an essential step for the understanding of the molecular machinery of the cell. Advances in structural genomics and proteomics [1-3] are producing a huge amount of information. Structural information is useful for inferring the protein biochemical function because often this function is strongly correlated with the protein 3D structure [4-6]. Moreover, structural properties can be useful for detecting a set of special amino acids that is strongly related to function. These amino acids are the protein functional residues, e.g, active site and binding site residues. Functional residues are the key players in protein-protein interactions and in protein-ligand interactions. Some recent studies have sought to find the common maximal set of functional residues for related proteins [7-14]. Those studies aimed at finding ligand functional sites may have a major impact on drug discovery development [15].

Protein-ligand interactions can be solved by assessing the co-crystallized ligand structure. Nowadays, there are several applications for this purpose. One such application is LigBase [16], a 3D ligand-binding database derived from the Protein Data Bank (PDB) [17]. In LigBase, a ligand-binding site is an amino acid with at least one atom within 5 Å from a ligand atom. There are many other applications based on the same principle, including PDBSUM [18], BindingDB [12], and PLD [13]. On the other hand, protein-protein interactions are hard to solve because large protein complexes are often more difficult to crystallize than protein-ligand complexes [19-22]. However, when functional residues of a protein family are known, they can be used to predict the function of related proteins. Identifying these residues from amino acid sequences alone would be useful for the understanding of the interactions of proteins with no 3D structure defined.

In order to predict functional sites in proteins with unknown function, the PROSITE database [23] uses a method based on regular expressions [24] (ScanProsite [25]) to detect functional site patterns and profiles within proteins with unknown function. In addition, Henschel and colleagues [22] presented a method based on hidden Markov models (HMM) [26-28] to predict functional sites in both protein-ligand and protein-protein interactions. Their research compiled sequential segments that constituted structural features of an interaction site into a hidden Markov model, namely the HMM descriptor. In their study, each Multiple Sequence Alignment (MSA) that represents a protein family generates fragments according to the position of functional residues, and for each fragment an HMM is built. Segmented HMMs are merged into one final HMM by their linking with insert states. This final HMM is called the HMM descriptor. Collection of HMM descriptors can be used to screen sequence databases by predicting functional site positions. Henschel and colleagues [22] focused on the protein-protein functional site predictions, but they also evaluated ligand-binding descriptors for the ATP-binding.

Herein, we present a method based on hidden Markov models (HMM) that uses functional sites of known ligand-protein interactions to predict functional sites in query sequences. Our method was based on the study done by Henschel et al [22], with some differences. First, we built the HMM from full MSAs rather than from functional sites of MSA segments. Second, HMMs for the prediction of functional residues were trained with both multiple sequence alignments based on structural alignments and with column alignments which corresponded to functional residue positions. Third, our approach focused on predictions of protein-ligand functional sites rather than predictions of protein-protein functional sites. The ligand-binding residues are likely to be important in many applications such as in designing small molecular inhibitors for drug discovery. Finally, we developed a web-accessible database, called **S**tructural **D**escriptor **D**ata**B**ase (SDDB) that uses our prediction method and allow the detection of functional sites, biological activity, and of ligand interactions in query sequences.

The SDDB was built with information from several databases, namely the PDB [17], SCOP [29], PDB-SUM [18], and CSA [30]. Each family in the SCOP database was associated with a library of HMM descriptors. We selected 1,902 SCOP families and 70,215 PDB entries. Functional sites were obtained from PDBSUM (binding-ligand residues) and from CSA (active-ligand residues). Structural alignments were derived from the 3DCOFFEE aligner [31]. We used the HMMER package [32] for building HMM descriptors. We also built a user friendly website to turn both the database and the functional site prediction method available to anyone interested . In this website, query sequences can be scored by using a library of HMM descriptors. The SCOP classification, functional residue positions, and ligand binding interactions are considered in order to indicate the sequence most likely function. Also, SDDB contains links to all related databases, which allows the user to have immediate access to additional information on the protein of interest.

## Results and discussion

In order to validate our prediction method, we performed experiments using three different data sets. We started by assessing one specific family, namely the Trypsion-like Serine proteases. Next, we assessed the remaining SCOP families. Finally, we compared our results with those on the ATP-binding study done by Henschel and colleagues [22]. In the first experiment, the functional residue positions were defined from protease study [33]. In the second and third experiments, the functional residue positions were extracted from the PDBSUM and the CSA databases. In the first and second experiments we wanted to assess our prediction method when both data was available, curated data supplied by expert knowledge and automated data calculated from computational methods, as done previously by others [34-36].

### Trypsion-like Serine proteases data set

In our first experiment, we only considered SCOP sequences from the Trypsion-like Serine protease family. We selected a total of 39 proteins. Sequences were separated into 8 groups, according to ligand specificity. These groups are Chymotrypsin, Elastase, Granzyme, Thrombin, Urokinase, Trypsin, Plasminogen Activator, and a hybrid group formed by Procarboxypeptidase A-S6 subunit III, Granzyme K, and Neuropsin. Table 1 shows for each group the PDB identify, chain, start residue and end residue that refer to the PDB coordinate file for each protein selected. Also, table 1 shows alignment identity for each group.

**Table 1:** Groups of Trypsion-like Serine protease.

Group name	PDB-Ids	Chain	Start Residue	End Residue	Alignment identity
Chymotrypsin	1gmc	A	1	245	52%
	1cho	E	1	245	
	1azz	A	16	246	
	1eq9	A	16	244	

Kallikrein	1ao5	A	16	246	32%
	1op8	A	16	246	
	1aut	C	16	243	
	1bqy	A	16	245G	
	1lo6	A	16	243	
	1eax	A	16	244	
	1m9u	A	16	242	

Granzyme	1euf	A	16	243	62%
	1iau	A	16	244	
	1fi8	A	16	244	

Thrombin	1bth	H	16	242	47%
	1id5	H	16	244	
	1etr	H	16	247	
	1fjs	A	16	244	
	1h8d	H	16	246	
	1fxy	A	16	246	
	1gvk	B	16	245	
	1pfx	C	16	245	
	1rfn	A	16	245	

Urokinase	1fiw	A	16	254	43%
	1fiz	A	16	257	
	1fv9	A	1	244	

Trypsin	1d6r	A	16	245	73%
	1mct	A	16	245	
	1slu	B	16	245	
	3tgi	E	16	245	
	1h4w	A	16	246	
	1hj8	A	16	245	
	1trn	A	16	246	

Plasminogen activator	1a5i	A	1A	244	46%
	1bda	A	1A	244	
	1gvz	A	16	246	

Hybrid group	1fon	A	9	240	29%
	1mza	A	14	248	
	1npm	A	16	243	

Group name, PDB coordinates details, such as PDB-Id, chain, start residue, and end residue of each protein used in first experiment. In addition, the later column shows the identity of alignment of each protein group.

We performed cross validation by applying the leaving one out method [37], in each group. First, training set sequences were aligned using the 3DCOFFEE tool [31]. Next, for each alignment, positions of binding site alignment and of active site alignment were defined, according to [33]. HMM descriptors were built using both alignments and alignment-columns, which corresponded to the residues of the functional sites. These HMM descriptors were used for screening and predicting functional residue positions in the test-sequence set.

Table 2 reports precision and recall values for each group, for e-values > 1. The Trypsion-like Serine protease experiment achieved an average of 99.61% of precision and 99.62% of recall, when predicting the active site. Regarding the binding site predictions, this experiment achieved an average of 98% of precision and 97% of recall. These results show that active site residues are more conserved than binding site residues. Therefore, HMM descriptors performed better when active sites were assessed. Also, we assessed protein function prediction by classifying test-sequence according to the SCOP database. As a result, 100% of the proteins were correctly classified into the SCOP class, fold, super family, and family. Additionally, 97.1% was classified into its correct domain.

**Table 2:** Matches for Trypsion-like Serine protease groups.

Group name	AS Precision	AS Recall	BS Precision	BS Recall
Chymotrypsin	97	98.8	97.8	96.8

Kallikrein	100	100	99	98.6

Granzyme	100	99.2	100	98.9

Thrombin	99.9	100	98	97.2

Urokinase	100	100	97.9	97.1

Trypsin	100	100	100	100

Plasminogen activator	100	99.8	98.5	97.3

hybrid group	100	99.2	96.1	96.3

Precison and recall for each group in Trypsion-like Serine protease data set

### SCOP data set

The second experiment was performed considering the SCOP families selected for this study (see Method section for election criterion), with the exception of the Trypsion-like Serine protease family. A total of 1,901 families were selected. Similarly as in the first experiment, we performed cross validation by applying the leave one out test [37]. Sequences in each family were separated into groups according to their ligand interactions. Training sequences were aligned using 3DCOFFEE [31]. Binding and active site alignment positions were obtained by running an algorithm that recognizes functional ligand residues in alignments, as explained in Methods. Next, HMM descriptors were built using both alignment and functional ligand positions. Finally, each HMM descriptor was used for screening, predicting, and classifying a test-sequence.

The SCOP families experiment achieved precision of 84% and recall of 70.8%, in predicting the active site. For binding site prediction, precision and recall were 78% and 57%, respectively. As observed in the first experiment, our methodology obtained better results in predicting active sites rather than in predicting binding sites. Indeed, active site residues are more conserved than binding residues. Therefore, we applied different levels of tolerance when defining an alignment column into a binding or active site. For instance, we set as binding site columns those that presented more than 50% of their binding residues aligned, whereas for active sites columns we required 80% of active residues aligned. These parameter settings narrowed the active site predictions because they decreased the number of false positives that could be incorporated in the training data, which in turn increases precision. The recall also increases because fewer cases of false negatives are incorporated in the active site column.

We assessed the classification with respect to the SCOP database, and found that all proteins were correctly classified into the SCOP class, fold, super family, and family. Around 96% of the proteins were classified into their correct domain.

### ATP-binding data set

We compared our results with those obtained by Henschel and colleagues [22]. For that, we assessed HMM descriptors performance for the particular study of the ATP-binding site. We considered 94 SCOP families in which a ATP-ligand or ADP-ligand was present, totalizing 10,520 sequences. Notice that their tests were performed on SWISS-PROT [38] database, and they used 36,774 sequences, while we used the SCOP database, a dataset smaller than Swiss-Prot. Unfortunately, direct comparisons are not possible because their source code is not available. For this experiment we assessed precision-recall for e-values < 1. Table 3 shows our results and those of Henschel and colleagues'. Their original results can be found in table 1[22]. They achieved a precision of 89.43%, whereas we achieved 86.39%. However, our results had a recall rate of 55.72%, whereas theirs was 25.17%. This indicates that our prediction method has produced fewer false negatives. While our method detected more false positives, it also detected more true positives.

**Table 3:** Matches for ATP-binding data set.

	Precision	Recall
Henschel et al	89.43	25.17

SDDB	86.39	55.72

Precison and recall for both Henschel and colleagues prediction method, and our approach (SDDB).

### Assessing HMM Descriptors performance

**Figure 1:**
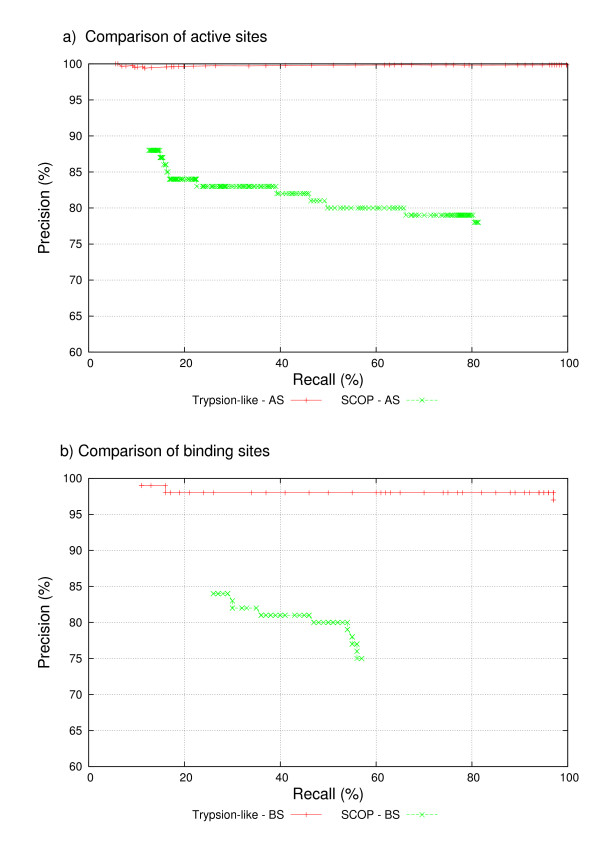
**Comparison of Trypsion-like Serine protease and SCOP descriptors through precision-recall curves**. SDDB performance for all data sets, as measured by precision-recall curves. Each point in the graphic corresponds to a different e-value cutoff. A – Active site prediction. B – Binding site prediction.

To evaluate the performance of HMM descriptors, we built precision-recall curves for our first and second experiments. Figure 1-a shows precision-recall curves for active site predictions, whereas figure 1-b reports precision-recall curves for binding site predictions. Table 4 shows protein function classification for both experiments. We noted that the first experiment performed better than the second one in all cases. We compared the experiment performance for active site prediction only, and we observed that the area under the graphic is greater for the Trypsion-like Serine protease experiment than for the SCOP family experiment (figure 1-a). Also, figures 1-b shows greater areas for the Trypsion-like Serine protease experiment. Results in the first experiment were expected because the training data set is a curated data, as they were collected from the literature. We believe, they are more reliable than computationally-calculated functional residues. This significant difference in the performance of these experiments may be due to separation into groups according to ligand specificity. Proteins from the same family can present different functional sites when interacting with the same ligand, i.e., the type of amino acids involved in interaction might not be the same ones. Thus, these proteins present different ligand specificity. Naturally, the alignment of proteins with similar ligand specificity keeps the functional sites aligned. As a result, HMM descriptors derived from these alignments perform better than those built by using alignments that contain all the proteins that interact with the same ligand, which explains the better results obtained in the first experiment. In any way, HMM Descriptor performed better achieved precision above 70% and recall above 50%, in both cases.

**Table 4:** SCOP classification.

	Class	Fold	Super Family	Family	Domain
Trypsion-like Serine protease	100	100	100	100	97.1

SCOP	100	100	100	100	96.2

SCOP classification for Trypsion-like Serine protease and SCOP data sets.

## Conclusion

With this study we aimed to develop a web-based tool that allowed the use of a computational method for the prediction of functional sites and protein function. For that, we created a prediction method based on the study of Henschel and colleagues [22] in which they developed a method for predicting functional sites in proteins with unknown function. Our web-based tool does two main tasks: it screens the SCOP database for the prediction of the protein function, and it pint-points the positions of its functional residues. Likewise the study of Henschel and colleagues, our prediction method is based on the hidden Markov model descriptors and structural properties. However, our HMMs were built using information from structural alignments, ligand binding sites, and active sites.

Our web-accessible database is a user friendly interface that allows users to analyze their own data. After setting the parameters, users can submit their protein sequences. Each protein is then analyzed and results are sent by email. Each sequence submitted is scanned against a library of HMM descriptors and results are displayed in two levels. In the first level of results, it shows all predicted functional residues and protein interactions with its ligands. In the second, it shows the predicted functional residues for each interacting ligand, individually. HMMs and alignments are available for download in the result page.

To validate our prediction method, we performed experiments that achieved significant precision-recall ranges. Our approach is accurate in determining protein-ligand interactions and interaction-residues positions. Therefore, SDDB is useful for functional annotation and for predicting functional residues. Knowing the positions of functional residues may provide insights into the biological activity of the protein and reveal new targets for drug development.

## Methods

The web-based tool was developed in Java version 1.5 and is now served through a Tomcat apache web server running on a Linux operating system. The web-site was built with servlets, JSP, and java scripts technologies. To store our data, we built a relational database manager using Postgres, version 8.2. The SDDB was populated with data extracted from public databases such as SCOP, version 1.71, PDB (downloaded in May, 2007), PDBSUM (downloaded in May, 2007) and CSA version 2.2.5. PDB is a repository for 3D structural data of proteins widely used in structural proteomic analysis. SCOP is a manually inspected database of protein folds that is particularly interesting to our study because it describes structural and evolutionary relationships between proteins, including all entries in PDB. PDBSUM is a database that provides, for each PDB file, a summary of the key structural features, including protein-ligand interaction and binding sites positions. Finally, CSA catalogs catalytic residues in enzymes of 3D structures. Based on the family level of SCOP, we extracted and classified the majority of protein-ligand interactions found in the PDB. HMMs were built from structural alignment derived from 3DCOFFEE, using HMMER package. This stage is the so-called training phase (see below). The Inference phase is the step in which query sequences are submitted to SDDB tool.

### Training Phase

In this phase data was fed into the SDDB to be later used for inference. Initially, we used other databases to obtain the necessary data such as SCOP families, PDB structure files, ligands, and functional ligand residues. Next, structural alignments were built from the SCOP protein group using 3DCOFFEE. Finally, HMMER was performed to build HMM descriptors from both structural alignments and from functional ligand residues.

#### Extracting data from external databases

Initially, we selected all proteins in each SCOP family, except for those proteins without either ligand interactions or shared ligand interactions. Also, we removed the proteins whose size *T *was not in the range [*M *- *M*/2, *M *+ *M*/2], where *M *is the average size of the sequences in its respective SCOP family. This step is necessary because some of the SCOP family sequences greatly vary in size, which results in alignments with many gaps. As a result, HMMs built from a gapped alignment do not recognize homologous proteins well. With this initial data processing we obtained 1,902 families with 70,215 sequences.

**Figure 2:**
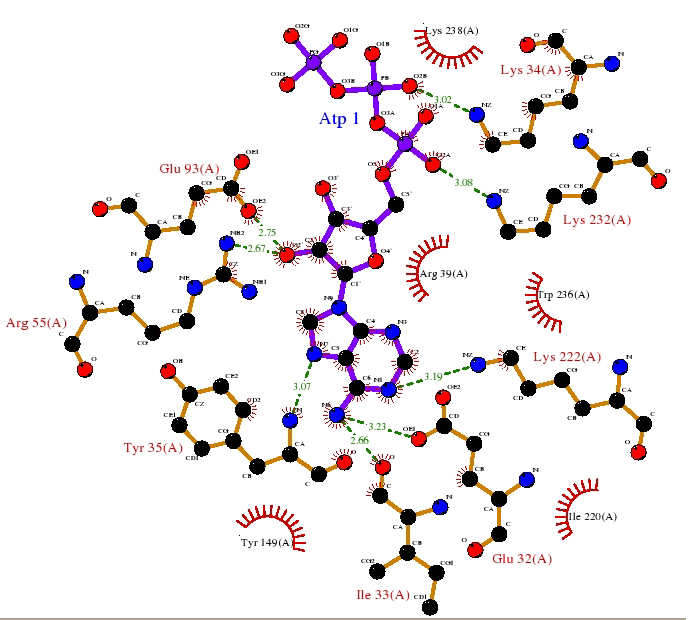
**LIGPLOT scheme for binding ligand residues**. The scheme shows the interaction between E. coli DNA ligase protein [40] and ATP (Adenosine-5'-triphosphate) ligand. In red are the DNA ligase's binding site residues.

Next, selected sequences were separated in groups according to the SCOP family level. For each sequence, we extracted from PDB its three-dimensional structure required for the structural alignment. From PDBSUM, we extracted the set of ligands that interact with each sequence and for each ligand we also extracted its protein binding sites. Figure 2 shows a LIGPLOT [39] scheme of the binding ligand residues (downloaded from PDBSUM) of the interaction between E. coli's DNA ligase (PDB id 1a0i) [40] and the ATP (Adenosine-5'-triphosphate) ligand. The active ligand residues of the interaction between SCOP protein and PDB ligand were extracted from the CSA database.

#### Building Structural Groups

**Figure 3:**
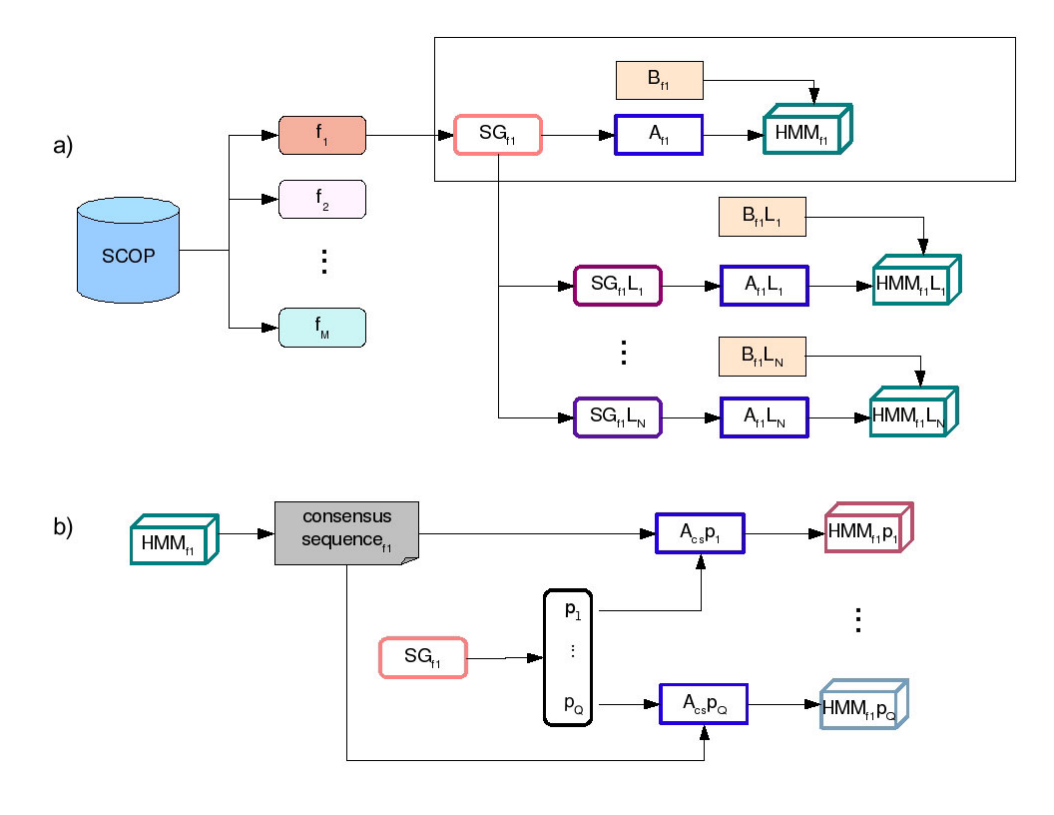
**Creating HMM descriptors from SCOP families**. Each SCOP family was segmented in structural groups and HMM descriptors were created from these groups. A – Image shows the building of HMM descriptors for a hypothetical family, namely f_1_. First, $A_{f_{1}}$ is built by aligning all proteins of $SG_{f_{1}}$ structural group. Next, ${\text{HMM}}_{f_{1}}$ is built from both $A_{f_{1}}$ alignment and functional site positions of $A_{f_{1}}$, called $B_{f_{1}}$. Finally, f_1 _is divided into groups of proteins that interact with the same ligand, and ${\text{HMM}}_{f_{1}}L_{i}$ are built in the same way, since *L *is the number of ligand and 1 ≤ *i *≤ *N*. B – In order, for building HMM classifications, the ${\text{consensus-sequence}}_{f_{1}}$ and each protein in $SG_{f_{1}}$ are aligned by producing *A*_*cs*_*P*_*i*_, where 1 ≤ *i *≤ *Q*. The building of ${\text{HMM}}_{f_{1}}p_{i}$ classificator is based on *A*_*cs*_*P*_*i*_.

Let *f *be an arbitrary SCOP family. All proteins of *f *make up the core structural group of this family, namely *SG*_*f *_(structural group of *f*). The alignment of *SG*_*f *_proteins was called *A*_*f *_and it was used as the starting point for the construction of the HMM descriptor that represents the *f*, called by HMM_*f*_. A family *f *is made up of a set of proteins *P *= {*p*_1_,...,*p*_*Q*_}, where *Q *is the number of sequences within an *f*. Each protein *p*_*i *_∈ *P *interacts with a set of ligands *L *= {*L*_1_,...,*L*_*n*_} downloaded from the PDBSUM. Each family *f *was divided into groups of proteins that interact with the same ligand. Then, a group was created only by proteins of *f *that interact with ligand *L*_*i*_. This group was called *SG*_*f*_*L*_*i *_(structural group formed by a subset of *f *that interacts with *L*_*i*_). Each *SG*_*f*_*L*_*i *_was defined as the side structural group. The alignment of *SG*_*f*_*L*_*i *_proteins was called *A*_*f*_*L*_*i*_. This alignment was the starting point for the construction of the HMM descriptors, namely HMM_*f*_*L*_*i*_. An HMM descriptor was built using both the MSA (*A*_*f *_or *A*_*f*_*L*_*i*_) and the alignment-columns, which corresponded to the residues of the functional sites (see below). Figure 3-a shows how each SCOP family was segmented in structural groups and how HMM descriptors were created from these groups.

#### Algorithm for recognizing functional ligand residues in alignments

The building of HMM descriptors required both the entire MSA and the columns referring to the amino acid alignment of the functional sites. Each protein *p *within the alignment has a set of amino acids that form, in regard to its interaction with a specific ligand, its functional sites, i.e. the ligand determines the set of functional sites of that protein. Because each protein *p *in *A*_*f*_*L*_*i *_has a set of functional sites associated to the *L*_*i *_interaction, we directly applied the algorithm for the identification of the functional sites. On the other hand, the *A*_*f *_alignment requires knowing the *cumulative sites *for all involved proteins, before applying the algorithm for the identification of functional sites. The cumulative sites of a protein *p *were calculated by combining sets of functional sites of the protein *p *with each of its ligands. Thus, if a protein *p *has interactions with ligands *L *= {*L*_1_,...,*L*_*j*_}, a set called *pL*_*k *_includes the positions of the functional sites that are associated to the interaction between *p *and *L*_*k*_, where *k *≤ *j*. As a result, the cumulative sites of a protein *p *that has the set of ligands *L *is created by *pL *= *pL*_1 _∪... ∪*pL*_*j*_.

After identifying the cumulative functional sites, we applied the algorithm described below. Each column in an MSA is individually assessed. Whenever 50% of the sequences contained a binding residue in that column, the column was marked as a binding site of the alignment. If 80% of the sequences contained an active residue at that column, then this column was marked as an active site of the alignment. Therefore, for each alignment *A*, there is a array *B*, with all elected columns of *A*, that represent functional site positions in *A*. Therefore, *B*_*f *_contained functional sites of proteins of *f *and *B*_*f*_*L*_*i *_contained functional sites of a subset of proteins of *f *that interacts with the *L*_*i *_ligand.

#### HMM construction

The HMM descriptors were built using an HMMER package. Given a *SG*_*f *_of a arbitrary SCOP family denoted by *f*, we built a HMM from *A*_*f *_and *B*_*f*_. We also built an HMM for each alignment in *SG*_*f*_*L*, where *L *is the set of ligands that interact with proteins of the *f*. Next, we built an HMM for each protein in *A*_*f *_and *A*_*f*_*L*_*i *_as follows. Each alignment contains a *P *= {*p*_1_,...,*p*_*n*_} protein subset, where each *p*_*i *_is a protein of the *f*, since *i *≤ *n *≤ *Q*. For each *p*_*i *_an HMM was built from its alignment with the consensus-sequence of the HMM that contains *p*_*i*_. The consensus-sequence is obtained from the alignment used to build the HMM, considering the most frequent amino acids in each column. These HMMs, called HMM classifications, were built to aid the SCOP classification of proteins of unknown function. Figure 3-b shows how to build HMM classifications for a hypothetical family *f*_1_. First, the consensus-sequence (called consensus-sequence *f*_1_) is extracted from ${\text{HMM}}_{f_{1}}$. Next, each protein *pi *in $SG_{f_{1}}$, where $SG_{f_{1}}$ is the structural group of *f*_1 _(see Building Structural Groups section), is aligned with consensus-sequence*f*_1 _through hmmalign program of HMMER package. This alignment is called *A*_*cs*_*p*_*i*_, where *p*_*i *_∈ $SG_{f_{1}}$ = {*p*_1_,...,*p*_*Q*_} and *Q *is the number of sequences in *f*_1_. Each *A*_*cs*_*p*_*i *_results in the building of ${\text{HMM}}_{f_{1}}p_{i}$.

### Inference Phase

After establishing and characterizing the training phase, the web-tool can then be used for predicting functional sites and protein function of user query sequences. Users can submit sequences in fasta format to the web site. Each sequence *p *is submitted to the HMM_*f*_, where *f *represents the families selected by the user. Let $\theta_{f_{p}}$ be e-value assigned to scoring of *p *by HMM_*f*_. If $\theta_{f_{p}}$ <*θ*_*f*_, *p *is accepted, i.e, it is likely that *p *is a member of the *f*. The *θ*_*f *_is the threshold necessary in order for *p *to belong to *f*. When *p *is accepted, the cumulative functional site positions and the *p *protein function are predicted, followed by *p *scoring by all HMM_*f*_*L*_*i*_, where *L*_*i *_∈ *L *and *L *is the set of all ligands that interact with proteins of *f*. Similarly, if $\theta_{fp}L_{i}$ <$\theta_{fL_{i}}$ then *p *is accepted, where $\theta_{fpL_{i}}$ is the e-value assigned to scoring of *p *by HMM_*f*_*L*_*i*_, and $\theta_{fL_{i}}$ is the threshold for considering *p *interacting with *L*_*i*_. When *p *is accepted, the functional sites of interaction between *p *and *L*_*i *_are predicted.

#### Predicting binding and active ligand residues

**Figure 4:**
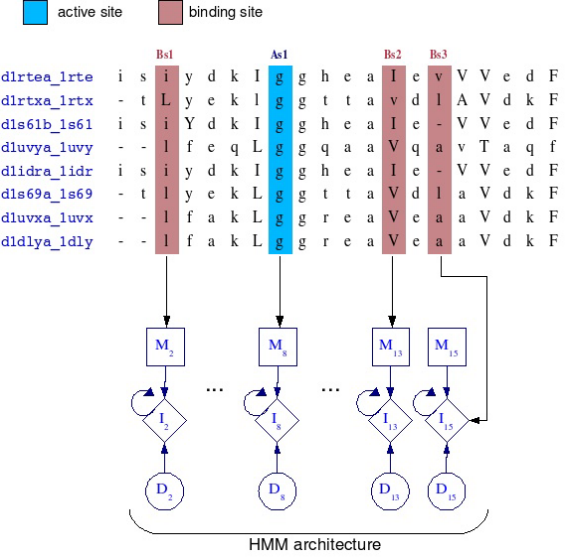
**Mapping functional residues to HMM states**. Each column in the globin alignment maps to either match or insert state, including the columns that represent functional sites. The columns labeled by *As *represents active site positions, whereas *Bs *columns represents binding site positions. *M*_*i*_, *I*_*i *_and *D*_*i *_represent match, insert and delete states in HMM architecture, respectively. In this illustration, As1 mapped to *M*_8 _state, and the *Bs*_1_, *Bs*_2 _and *Bs*_3 _columns mapped to *M*_2_, *M*_13 _and *I*_15 _states, respectively.

The alignment-columns containing functional sites were mapped to the HMM states, i.e, either match or insert states, as shown in figure 4. The figure shows a partial alignment of proteins for the globin family, in which the column labeled *Bs*_1 _that represents a binding site position in the alignment mapped to the match state *M*_2_. Similarity, the column labeled *As*_1_, which represents an active site position in the alignment, mapped to the match state *M*_8_. Note that the column labeled *Bs*_3 _mapped an insert state *I*_15_. Hence all columns in the alignment were represented by one HMM state. When a protein *p *is scored by an HMM, the Viterbi algorithm [41] provides both e-value and the best path found involving scored *p *amino acids through the HMM architecture. This path is the sequence of states by which the *p *amino acids were recognized for *p *to be classified by the HMM. We knew which of the states of the HMM represented the functional sites (figure 4), so we were able to determine whether the amino acids of *p *were recognized by those states, and thus predict the positions of the functional sites of *p*. For instance, let *p *= (*a*_1_, *a*_2_,...,*a*_*n*_) be a protein sequence, where *a*_*i *_is a amino acid in the *i *position, and let HMM_*f *_be an HMM that represents an arbitrary family *f*, then we are interested in *Pr*[*p*|HMM_*f*_], which describes the probability of observing a protein *p *within HMM_*f*_. The Viterbi algorithm give us both *Pr*[*p*|HMM_*f*_] and *π*, where *π *is the best path of *p *through HMM_*f *_states. If *π *is given by *M*_1_*M*_2_*I*_3_...*M*_*n*_, then it means that *a*_1 _was recognized by the match state *M*_1_, *a*_2 _was recognized by the *M*_2_, *a*_3 _was recognized by the insert state *I*_3_, until *a*_*n *_was recognized by *M*_*n*_. Therefore, if *M*_2 _is a state that represents a binding site, it is likely that *a*_2 _in *p *is a binding site residue.

#### Annotating query sequences

After submitting a *p *protein to an HMM descriptor and verifying that its e-value is smaller than *θ*_*f*_, it is necessary to determine the following parameters for the SCOP classification: class, fold, super family, family, and domain. We annotated *p *by comparing it with all HMM classifications (see section HMM construction) and by associating to *p *the same classification of *p*_*i*_, where *p*_*i *_is the protein most similar to *p*, and which belongs to the HMM classification. For instance, suppose that some protein *p *has been scored by ${\text{HMM}}_{f_{1}}$ as in figure 3-b. Also, consider that *p *has been classified with e-value $\theta_{f_{1p}}$, where $\theta_{f_{1p}}$ ≤ $\theta_{f_{1}}$. Next, *p *is scored by each ${\text{HMM}}_{f_{1}}p_{i}$, where *p*_*i *_∈ {*p*_1_,...,*p*_*Q*_} and *Q *is the number of sequences in *f*_1_. Each ${\text{HMM}}_{f_{1}}p_{i}$ assigns a $\theta_{f_{1}p_{i}}$ e-value to *p*. Suppose $\theta_{f_{1}p_{1}}$ <$\theta_{f_{1}p_{2}}$ < ... <$\theta_{f_{1}p_{n}}$, thus *p *receives the SCOP classification of *p*_1_.

## Authors' contributions

JSB performed the studies and participated in writing the manuscript. JHF participated in the design of the study and ATRV participated in the design and coordination of the study, and in writing the manuscript. All authors read and approved the final manuscript.

## References

[B1] Chandonia J, Brenner S (2006). The impact of structural genomics: expectations and outcomes. Science.

[B2] Bateman A, Valencia A (2006). Structural genomics meets computational biology. Bioinformatics.

[B3] Kim S, Shin D, Choi I, Gahmen U, Chen S, Kim R (2003). Structure-based functional inference in structural genomics. J Struct Funct Genomics.

[B4] Watson J, Laskowski R, Thornton J (2005). Predicting protein function from sequence and structural data. Current opinion in structural biology.

[B5] Baker E, Arcus V, Lott J (2003). Protein structure prediction and analysis as a tool for functional genomics. Applied bioinformatics.

[B6] Baker D, Sali A (2001). Protein structure prediction and structural genomics. Science.

[B7] Polacco B, Babbitt P (2006). Automated discovery of 3D motifs for protein function annotation. Bioinformatics.

[B8] Goyal K, Mohanty D, Mande S (2007). PAR-3D: a server to predict protein active site residues. Nucleic Acids Res.

[B9] Nebel J, Herzyk P, Gilbert D (2007). Automatic generation of 3D motifs for classification of protein binding sites. BMC Bioinformatics.

[B10] Kinoshita K, Murakami Y, Nakamura H (2007). eF-seek: prediction of the functional sites of proteins by searching for similar electrostatic potential and molecular surface shape. Nucleic Acids Res.

[B11] Shin J, Cho D (2005). PDB-Ligand: a ligand database based on PDB for the automated and customized classification of ligand-binding structures. Nucleic Acids Res.

[B12] Chen X, Liu M, Gilson M (2001). BindingDB: A Web-Accessible Molecular Recognition Database. Combinatorial Chemistry & High Throughput Screening.

[B13] Puvanendrampillai D, Mitchell J (2003). Protein Ligand Database (PLD): additional understanding of the nature and specificity of protein ligand complexes. Bioinformatics.

[B14] Okuno Y, Yang J, Taneishi K, Yabuuchi H, Tsujimoto G (2006). GLIDA: GPCR-ligand database for chemical genomic drug discovery. Nucleic Acids Res.

[B15] Campbell S, Gold N, Jackson R, Westhead D (2003). Ligand binding: functional site location, similarity and docking. Current Opinion in Structural Biology.

[B16] Stuart A, Ilyin V, Sali A (2002). LigBase: a database of families of aligned ligand binding sites in known protein sequences and structures. Bioinformatics.

[B17] Helen M, Westbrook J, Feng Z, Gilliland G, Bhat T, Weissig H, Shindyalov I, Bourne P (2000). The Protein Data Bank. Nucleic Acids Research.

[B18] Laskowski R, Chistyakov V, Thornton J (2005). PDBsum more: new summaries and analyses of the known 3D structures of proteins and nucleic acids. Nucleic Acids Res.

[B19] Dohkan S, Koike A (2003). Support Vector Machines for Predicting Protein-Protein Interactions. Genome Informatics.

[B20] Farisellil P, Zauli A, Rossi I, Finell M, Martelli P, Casadio R (2003). A neural network method to improve prediction of protein-protein interaction sites in heterocomplexes. XI11 Workshop on Neural Networks for Signal Processing, IEEE.

[B21] Tran T, Satou K, Ho T (2005). Using Inductive Logic Programming for Predicting Protein-Protein Interactions from Multiple Genomic Data. Knowledge Discovery in Databases: PKDD.

[B22] Henschel A, Winter C, Kim W, Schroeder M (2007). Using structural motif descriptors for sequence-based binding site prediction. BMC Bioinformatics.

[B23] Hulo N, Bairoch A, Bulliard V, Cerutti L, Cuche B, Castro E, Lachaize C, Langendijk-Genevaux P, Sigrist C (2007). The 20 years of PROSITE. Nucleic acids research.

[B24] Hofmann K (2000). Sensitive protein comparisons with profiles and hidden Markov models. Brief Bioinform.

[B25] Castro E, Sigrist C, Gattiker A, Bulliard V, Langendijk-Genevaux P, Gasteiger E, Bairoch A, Hulo N (2006). Scan-Prosite: detection of PROSITE signature matches and ProRule-associated functional and structural residues in proteins. Nucleic acids research.

[B26] Rabiner L (1989). A Tutorial on Hidden Markov Models and Selected Applications in Speech Recognition. Proceedings of the IEEE.

[B27] Eddy S (1996). Hidden markov models. Current Opinion in Structural Biology.

[B28] Krogh A, Brown M, Mian I, Sjolander K, Haussler D (1994). Hidden markov models in computational biology applications to protein modeling. Journal of Molecular Biology.

[B29] Andreeva A, Howorth D, Brenner S, Hubbard T, Chothia C, Murzin A (2004). SCOP database in 2004: refinements integrate structure and sequence family data. Nucleic Acids Research.

[B30] Porter C, Bartlett G, Thornton J (2004). The Catalytic Site Atlas: a resource of catalytic sites and residues identified in enzymes using structural data. Nucleic Acids Research.

[B31] Sullivan O, Suhre K, Abergel C, Higgins D, Notredame C (2004). 3DCoffee: combining protein sequences and structures within multiple sequence alignments. Journal of Molecular Biology.

[B32] Eddy S (1998). Profile hidden Markov models. Bioinformatics.

[B33] Fernandez J, Mello M, Galgaro L, Tanaka A, Silva-Filho M, Neshich G (2007). Proteinase inhibition using small Bowman-Birktype structures. Genet Mol Res.

[B34] Keunwan P, Dongsup K (2006). A Method to Detect Important Residues Using Protein Binding Site Comparison. Genome Informatics.

[B35] Ferre F, Ausiello G, Zanzoni A, Helmer-Citterich M (2005). Functional annotation by identication of local surface similarities: A novel tool for structural genomics. BMC Bioinformatics.

[B36] Shulman-Peleg A, Nussinov R, Wolfson H (2004). Recognition of functional sites in protein structures. Journal of Molecular Biology.

[B37] Mitchell T (1997). Machine Learning.

[B38] Bairoch A, Apweiler R (1997). The SWISS-PROT protein sequence database: its relevance to human molecular medical research. Journal of molecular medicine.

[B39] Wallace A, Laskowski R, Thornton J (1995). LIGPLOT: A program to generate schematic diagrams of protein-ligand interactions. Protein Engineering.

[B40] Dunna J, Studiera F, Gottesmana M (1983). Complete nucleotide sequence of bacteriophage T7 DNA and the locations of T7 genetic elements. J Mol Biol.

[B41] Baldi P, Brunak S (2001). Bioinformatics: The Machine Learning Approach.

